# Focus on properties and applications of perovskites

**DOI:** 10.1088/1468-6996/16/2/020301

**Published:** 2015-03-27

**Authors:** Fatih Dogan, Hong Lin, Maryline Guilloux-Viry, Octavio Peña

**Affiliations:** 1 Missouri University of Science and Technology, USA; 2 Tsinghua University, People’s Republic of China; 3 University of Rennes 1, France

Perovskite materials with the same crystal structure as CaTiO_3_ exhibit intriguing and unusual physical properties that have been extensively studied for both practical applications and theoretical modeling. From the discovery of ceramic high-temperature superconductors to the organic–inorganic semiconductors for high-efficiency photovoltaics, the materials science and applications of perovskites have been a very broad research area open to many revolutionary discoveries for new device concepts. The impressive range of structure and property interplay of perovskites makes them an excellent research field for studies in materials science, physics and solid state chemistry. Structurally, such materials form various crystals including oxide and organometallic perovskites, spinels, and pyrochlores, to name just a few. Many different types of lattice distortions can occur owing to the flexibility of bond angles within the ideal perovskite structure. A broad range of novel functional materials and device concepts can be envisaged through fundamental understanding of the relationships between the structural and chemical compatibility, thermal stability, solid solubility and lattice strain.

In this focus issue of *Science and Technology of Advanced Materials*, we present articles that cover a wide range of topics on perovskites, which include ferroelectric, dielectric, pyroelectric, piezoelectric, magnetic, catalytic, photovoltaic and electronic conduction properties. While this focus issue may not exhaust the many aspects of perovskite materials, it does highlight our fundamental understanding and underscore many exciting developments in this field. We hope that these articles will stimulate more interest in perovskites and help readers better understand some important scientific and technological issues related to perovskite materials.

Special thanks are due to the authors and manuscript reviewers. Without their efforts, this focus issue would not have been completed.

